# Evaluation of recent lightweight deep learning architectures for lung cancer CT classification

**DOI:** 10.3389/fonc.2025.1647701

**Published:** 2025-09-19

**Authors:** MennaAllah Mahmoud, Yanhua Wen, Xiaohuan Pan, Yuling Liufu, Yubao Guan

**Affiliations:** ^1^ Radiology Department, The Fifth Affiliated Hospital of Guangzhou Medical University, Guangzhou, China; ^2^ Radiology Department, First Affiliated Hospital of Guangzhou Medical University, Guangzhou, China

**Keywords:** deep learning, pre-trained models, lightweight models, lung cancers, computed tomography

## Abstract

**Introduction:**

While numerous large and complex deep learning architectures continue to be developed for medical imaging, clinical adoption remains limited to a small number of established models. Recent lightweight architectures, despite showing promise in computer vision tasks, are underutilized or have never been applied to medical imaging applications, particularly lung cancer classification. This study evaluates the performance of recently developed lightweight models that have received limited attention in medical imaging tasks, establishing comprehensive baseline comparisons to guide evidence-based selection for clinical deployment in resource-constrained environments.

**Methods:**

Using CT images, we assessed three lightweight pre-trained models—MobileOne-S0, FastViT-S12, and MambaOut-Femto for lung cancer categorization. Performance measures (accuracy, AUC) and efficiency measures (inference time, number of parameters) were contrasted. Used were a public dataset (95 cases) and a private dataset (274 cases). Resampling and data augmentation constituted part of image preparation. Five-fold cross-validation helped to validate model performance.

**Results:**

With the lowest inference time and modest parameters, MambaOut-Femto displayed the best efficiency. While FastViT-S12 had the largest memory usage, MobileOne-S0 used fewer parameters. On Dataset 1, MambaOut-Femto obtained a mean accuracy of 0.896 ± 0.014 and an (Area under the curve) AUC of 0.972 ± 0.004; on Dataset 2, accuracy was 0.916 ± 0.040. When compared to traditional models like ResNet and Swin Transformer on the same datasets and under the same hyperparameters, the lightweight models outperformed them with significantly lower memory usage and fewer FLOPs.

**Discussion:**

The lightweight models demonstrated superior efficiency and comparable performance to traditional models, making them ideal for deployment in low-resource settings where computational resources are limited. These findings highlight the potential for practical use in clinical workflows, overcoming barriers associated with traditional models.

## 1 Introduction

Lung cancer continues to be the most common cause of cancer-related death globally, with adenocarcinoma and squamous cell carcinoma accounting for over 85% of cases (1, 2).

Cancer diagnosis has been radically altered by the incorporation of artificial intelligence (AI) and computer-aided diagnosis (CAD) systems, which represent an enormous move forward from conventional radiomics to complex deep learning methods. Deep learning algorithms have shown exceptional skills in automated feature detection and multi-lesion tracking, frequently outperforming humans in certain diagnostic tasks, thanks to these technical advancements that have overcome several limitations in traditional approaches (3).

However, despite these major achievements, some substantial implementation obstacles exist in clinical practice. High processing demands, intricate integration specifications with current systems, restricted interpretability, and reproducibility of results are some of these difficulties (3, 4). The broad adoption of these technologies is further hampered by practical limitations like restricted data availability due to privacy regulations, a lack of standardized implementation protocols, and difficulties integrating them with current clinical workflows (5, 6).

Recent advances in lightweight architectures have introduced several promising solutions to address these challenges. Lightweight Convolutional Neural Networks (CNNs) such as MobileOne, TinyNet, LCNet, and GhostNetV2 have been designed to optimize network scaling, efficient inference, and hardware-specific accelerations, making them suitable for deployment in resource-constrained environments (7–10). Similarly, efficient transformers like EfficientFormer, RepViT, FastViT, and Mamba have addressed memory and computational limitations, offering high efficiency and performance gains through innovative designs and hybrid approaches (11–15).

Previous studies have shown success with lightweight architectures in medical imaging, specifically for lung cancer classification and detection. For instance, Attallah et al. (2022) proposed a framework for lung and colon cancer diagnosis using lightweight deep learning models and transformation methods, achieving a high accuracy of 99.6% (16). Priya and Shyamala Bharathi (2024) demonstrated that a deep learning-based pre-trained model called EfficientNet achieved 99.28% training accuracy and 98.03% testing accuracy for lung cancer detection and classification using CT images (17). Cao et al. (2023) developed a multi-scale mobile-based model (18), the proposed model achieves comparable or superior performance while maintaining a more lightweight architecture. These studies highlight the potential of lightweight models in maintaining high diagnostic accuracy while reducing computational complexity.

Despite rapid advances in deep learning architectures, clinical adoption in medical imaging remains limited to established models, while recent lightweight architectures remain largely unexplored in healthcare applications. This creates a critical gap between research innovation and practical clinical implementation. Healthcare institutions require evidence-based comparative data to guide technology adoption decisions, particularly for resource-constrained environments where computational efficiency is paramount.

In this study, we aim to establish comprehensive baseline performance metrics for three recently developed lightweight pretrained models—MobileOne-S0, FastViT-S12, and MambaOut-Femto (19)—that have received limited attention in medical imaging applications. We systematically compare their efficiency and performance for lung cancer CT scan image classification to provide evidence-based guidance for clinical deployment decisions.

## 2 Materials and methods

### 2.1 Model selection

In this study, we conducted a comprehensive exploration of different lightweight pretrained models that meet the criteria to be lightweight, released in or after 2023, have pretrained weights, and architectures that have been underutilized or not previously applied to medical image classification, specifically for the task of lung cancer CT scan image classification. The selection process involved a comprehensive search of pretrained models available in various deep learning libraries, including Keras, PyTorch’s timm, and Huggingface.

To identify suitable candidates, we employed both manual sorting and AI-assisted tools to compile a list of models meeting our predefined criteria. Subsequently, we conducted an extensive literature review using Google Scholar and PubMed incorporating model names along with relevant keywords such as “lung cancer,” “classification,” and “CT scans”. This thorough search process enabled us to identify three promising lightweight architectures for in-depth examination:

MobileOne, FastViT, and MambaOut.

For each selected architecture, we opted to utilize the smallest recent variant, as these typically demonstrate superior performance characteristics. Consequently, the following specific model versions were chosen for our study: MobileOne-S0, Fastvit-s12, and MambaOut-Femto.

We quantitatively defined “lightweight” models using two key criteria (1): parameter count <10M and (2) activation memory < 15M. With MobileOne-S0 included, despite slightly exceeding the activation threshold due to its exceptional parameter efficiency, these thresholds were established based on model card specifications and represent practical computational constraints for deployment in resource-limited clinical environments where memory and processing capabilities may be constrained. This definition guided our systematic selection of the smallest available variants within each architecture family. The selected models met our lightweight criteria, with MobileOne-S0 containing 5.3M parameters and 15.5M activations, MambaOut-Femto with 7.3M parameters and 8.3M activations, and FastViT-S12 with 9.5M parameters and 13.7M activations. To contextualize these choices, even the smallest available transformer variant (SwinV2-tiny) contained 28M parameters and 28.5M activations, nearly double our lightweight threshold, demonstrating the computational efficiency advantages of our selected architectures for clinical deployment scenarios.

### 2.2 Dataset

Dataset1 is a private dataset from Institution 1 that contributed 274 cases (936 images), a non-enhanced CT dataset, which contains 119 cases (377 images) of adenocarcinoma (ADC), 93 cases (357 images) of benign lesions, and 62 cases (200 images) of squamous cell carcinoma (SCC).

Dataset2: a public dataset from Zenodo (Jian et al., 2024) contributed 95 cases (308 images), comprising 172 ADC images, 103 benign images, and 33 SCC images (20).

#### 2.2.1 Cross-dataset validation design

To evaluate model generalizability across different imaging protocols and patient populations, each dataset was acquired using distinct CT scanner configurations and represents different institutional practices. Dataset 1 (Institution 1) utilized Siemens Definition AS+ (128-slice and 64-slice) scanners with standardized protocols, while Dataset 2 (Jian dataset) incorporated multiple manufacturers (GE, Siemens, UIH) with varying slice configurations and convolution kernels (B70f, B60f).

#### 2.2.2 Image processing

For dataset1, preprocessing DICOM images in 3D Slicer (21) involved resampling the images to 1mm thickness with 512x512 pixel resolution and adjusting window settings to lung-specific parameters (width -600 to 1500 HU), then generating 2D images representing different locations of each nodule saved in PNG format.

Where dataset 2 was obtained directly in BMP-format images from the Zenodo repository.

Identical augmentation protocols were applied to both datasets to ensure consistency, including random horizontal flips (probability = 0.5), random rotations ( ± 15 degrees), color jitter (brightness=0.2, contrast =0.2, saturation =0.2, hue =0.1), resizing to 224×224 pixels (256×256 for FastViT-S12), and ImageNet normalization (mean=[0.485, 0.456, 0.406], std =[0.229, 0.224, 0.225]).

### 2.3 Model architectures and training

In our experiments, we employed the Mambaout femto, mobileone-s0, and FastViT-S12 model architectures with pretrained weights, fine-tuned for our specific task.

For data preparation, images were organized into subfolders within the dataset directory, with each subfolder representing a class.

Eight hyperparameter configurations were systematically evaluated using a held-out method (70% train, 15% validation, 15% test split). The configurations varied in batch size [16, 32], dropout rate (0.3, 0.5), optimizer (AdamW, RAdam), and weight decay (0.01, 0.1), while maintaining a fixed learning rate (0.0001). Each configuration was trained independently, with validation AUC used for early stopping and learning rate scheduling during training. After training all eight configurations separately, the configuration achieving the highest test set performance metrics was selected and subsequently applied consistently across all 5-fold cross-validation experiments without further tuning within individual folds.

In the second experiment, we utilized stratified 5-fold cross-validation to ensure balanced class distribution across folds, with each fold further split into training (80%) and validation (20%) sets, and a separate test set from the remaining fold. The best configuration that was determined from the first experiment was used across all folds.

Both experiments employed early stopping criteria based on validation AUC (patience of 10 epochs, minimum delta of 0.001) and utilized the ReduceLROnPlateau learning rate scheduler with a factor of 0.1 and a patience of 5 epochs. Training was conducted for up to 100 epochs per configuration or fold. Evaluation metrics included AUC (primary metric), accuracy, recall, specificity, and per-class metrics. The first experiment aimed to identify the optimal hyperparameter configuration based on validation AUC, while the second experiment focused on robustness evaluation through cross-validation, reporting average metrics across folds along with standard deviations. Visualization of results included learning curves, ROC curves, and confusion matrices for comprehensive performance assessment.

Each dataset was trained and validated independently using the held out method for model optimization and 5-fold cross-validation, with performance metrics reported separately. This approach provides a robust assessment of cross-domain performance under different scanner vendors, acquisition protocols, and patient demographics, demonstrating a model generalizability beyond single-institution data.

All experiments used a random seed of 42 to ensure reproducible results across dataset splitting, model initialization, and training procedures.

### 2.4 Benchmarking to other models

Using Dataset 1, with input sizes of 224×224 for most models and 256×256 for FastViT-S12, the dataset was split into training (70%), validation (15%), and test (15%) sets using random splitting with a fixed seed (1011) for reproducibility. Each configuration was trained five times with different random seeds (42, 456, 789, 1011, 2025) across separate experimental runs, enabling statistical analysis with 95% confidence intervals. All models were trained with a fixed configuration to ensure a fair comparison, featuring a batch size of 16, a dropout rate of 0.3, the AdamW optimizer with a weight decay of 0.1, and a learning rate of 0.0001. We employed the same early stopping criteria (patience of 10 epochs, minimum delta of 0.001 based on validation AUC) and ReduceLROnPlateau scheduler (factor 0.1, patience of 5 epochs). Mixed precision training was utilized to enhance efficiency.

We compared seven architectures, including MobileOne-S0, MambaOut-Femto, FastViT-S12, EfficientNet-B0 (22), ResNet-50 (23), ViT-Tiny (24, 25), and SwinV2-CR-Tiny (26), all initialized with pretrained weights and fine-tuned for the task.

#### 2.4.1 Comprehensive efficiency metrics

To align with clinical deployment needs for portable devices, we implemented comprehensive energy consumption profiling, including GPU power monitoring using the pynvml library for real-time wattage measurement, FLOPs calculation using ptflops for computational complexity assessment with fallback approximation methods when ptflops is unavailable, and enhanced memory profiling with proper cache management and peak allocation tracking.

All efficiency measurements were conducted with proper GPU memory isolation using torch.cuda.empty_cache() and torch.cuda.reset_peak_memory_stats() to measure true peak allocation. Inference timing included 20-iteration warm-up followed by 100-iteration measurement with GPU synchronization for accurate latency assessment. Statistical analysis was performed across all experimental runs, with metrics reported as mean ± standard deviation with 95% confidence intervals.

Comprehensive energy profiling on Tesla T4 GPU (16GB VRAM) on the Google Colaboratory cloud platform enables a fair comparison of computational requirements across architectures. The relative efficiency rankings provide guidance for model selection in deployment scenarios with computational constraints.

### 2.5 Model interpretability visualization

After determining the best-performing model, additional visualization analysis was conducted on random cases from each class (adenocarcinoma, squamous cell carcinoma, and benign) to demonstrate how the model makes diagnostic decisions. Preliminary testing across multiple network layers identified stages.1.blocks.0.conv as the optimal layer showing the strongest correspondence between AI attention patterns and pathological features. Custom activation extractors using PyTorch hooks capture feature responses at this selected layer. Activation maps were normalized to [0,1] range and visualized using a three-panel format: original CT scan, activation heatmap, and overlay visualization. All visualizations were generated using Matplotlib, OpenCV, and NumPy libraries.

### 2.6 Software and libraries

All experiments were implemented in a Python 3 environment using the Google Colaboratory cloud platform connected to a Tesla T4 GPU (16GB VRAM).

The implementation utilized the PyTorch framework with the timm library for pre-trained model architectures, torchvision for data transformations, scikit-learn for evaluation metrics, NumPy for numerical computations, Matplotlib for visualizations, pandas for data management and results analysis, and Seaborn for enhanced statistical visualizations.

## 3 Results

### 3.1 Efficiency metrics and benchmarking to common models

Among lightweight architectures, when tested on the same hyperparameters and dataset, MambaOut-Femto demonstrated superior performance with an accuracy of 91.06 ± 1.85% (95% CI [88.77-93.36%]) and AUC of 98.06 ± 1.17% (95% CI [96.61-99.51%]), substantially outperforming traditional architectures. In contrast, ResNet50, despite its widespread clinical adoption and 23.51M parameters, achieved only 72.34 ± 3.65% accuracy (95% CI [67.81-76.87%]) and 88.70 ± 2.28% AUC (95% CI [85.86-91.53%]), representing a 18.72% accuracy deficit compared to MambaOut-Femto. Similarly, SwinV2-CR-Tiny, with 27.57M parameters, showed disappointing performance with 70.21 ± 9.30% accuracy (95% CI [58.66-81.76%]) and 86.18 ± 6.11% AUC (95% CI [78.60-93.77%]).

**Figure 1:**
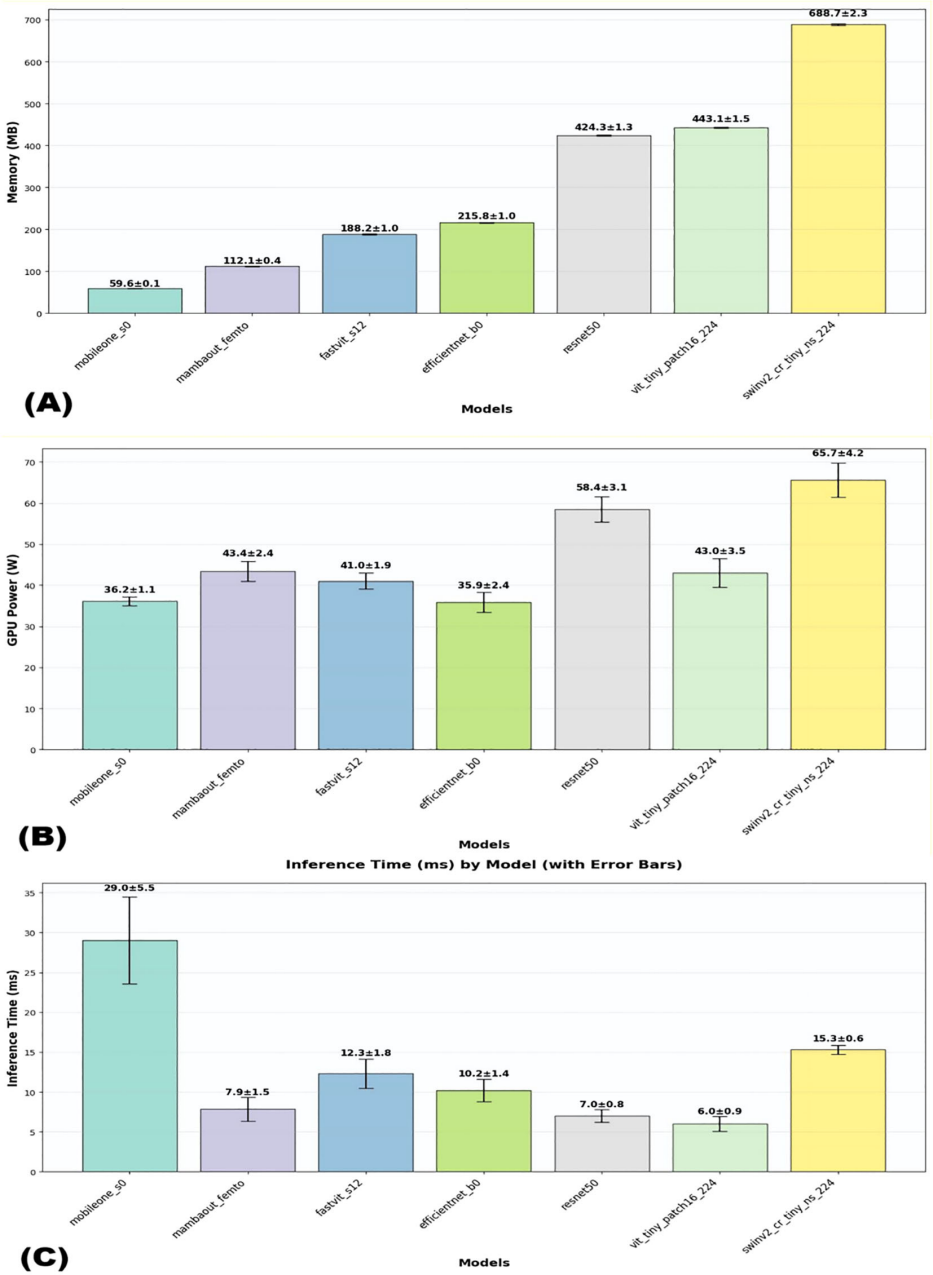
Visual comparison of efficiency metrics across models on dataset1. **(A)** Memory usage, **(B)** GPU usage, and **(C)** Interference time.

The efficiency metrics, including inference time, number of parameters, and memory usage, were compared across all models. As shown in **Figure 1** and detailed in **Table 1**,

**Table 1:** Comparative analysis of the models’ efficiency and performance across dataset1.

Model/Metrices	mobileone_s0	mambaout_femto	fastvit_s12	efficientnet_b0	resnet50	vit_tiny_patch16_224	swinv2_cr_tiny_ns_224
Inference Time (Mean ± Std) [ms]	29.029 ± 5.474	7.851 ± 1.473	12.320 ± 1.840	10.192 ± 1.397	6.991 ± 0.805	6.028 ± 0.938	15.317 ± 0.555
Inference Time 95% CI [ms]	[22.231, 35.826]	[6.023, 9.680]	[10.035, 14.606]	[8.458, 11.927]	[5.992, 7.990]	[4.863, 7.192]	[14.628, 16.006]
Parameters [M]	4.27	6.15	8.45	4.01	23.51	5.52	27.57
FLOPs (Mean ± Std) [G]	0.010 ± 0.000	0.010 ± 0.000	0.020 ± 0.000	0.010 ± 0.000	0.050 ± 0.000	0.010 ± 0.000	0.060 ± 0.000
FLOPs 95% CI [G]	[0.010, 0.010]	[0.010, 0.010]	[0.020, 0.020]	[0.010, 0.010]	[0.050, 0.050]	[0.010, 0.010]	[0.060, 0.060]
Memory (Mean ± Std) [MB]	59.6 ± 0.1	112.1 ± 0.4	188.2 ± 1.0	215.8 ± 1.0	424.3 ± 1.3	443.1 ± 1.5	688.7 ± 2.3
Memory 95% CI [MB]	[59.5, 59.7]	[111.6, 112.5]	[187.0, 189.4]	[214.6, 217.0]	[422.6, 425.9]	[441.1, 445.0]	[685.8, 691.6]
GPU Power (Mean ± Std) [W]	36.16 ± 1.06	43.43 ± 2.43	41.03 ± 1.90	35.88 ± 2.38	58.44 ± 3.10	42.95 ± 3.49	65.65 ± 4.16
GPU Power 95% CI [W]	[34.84, 37.48]	[40.41, 46.44]	[38.67, 43.38]	[32.92, 38.84]	[54.59, 62.28]	[38.62, 47.28]	[60.48, 70.81]
Accuracy (Mean ± Std)	0.8752 ± 0.0282	0.9106 ± 0.0185	0.9078 ± 0.0112	0.9078 ± 0.0241	0.7234 ± 0.0365	0.8241 ± 0.0406	0.7021 ± 0.0930
Accuracy 95% CI	[0.8402, 0.9102]	[0.8877, 0.9336]	[0.8939, 0.9217]	[0.8779, 0.9377]	[0.6781, 0.7687]	[0.7738, 0.8745]	[0.5866, 0.8176]
AUC (Mean ± Std)	0.9720 ± 0.0085	0.9806 ± 0.0117	0.9771 ± 0.0094	0.9789 ± 0.0056	0.8870 ± 0.0228	0.9406 ± 0.0232	0.8618 ± 0.0611
AUC 95% CI	[0.9615, 0.9826]	[0.9661, 0.9951]	[0.9653, 0.9888]	[0.9719, 0.9858]	[0.8586, 0.9153]	[0.9117, 0.9694]	[0.7860, 0.9377]
Sensitivity (Mean ± Std)	0.8752 ± 0.0282	0.9106 ± 0.0185	0.9078 ± 0.0112	0.9078 ± 0.0241	0.7234 ± 0.0365	0.8241 ± 0.0406	0.7021 ± 0.0930
Sensitivity 95% CI	[0.8402, 0.9102]	[0.8877, 0.9336]	[0.8939, 0.9217]	[0.8779, 0.9377]	[0.6781, 0.7687]	[0.7738, 0.8745]	[0.5866, 0.8176]
Specificity (Mean ± Std)	0.9318 ± 0.0150	0.9511 ± 0.0096	0.9494 ± 0.0069	0.9496 ± 0.0124	0.8482 ± 0.0193	0.9038 ± 0.0234	0.8346 ± 0.0534
Specificity 95% CI	[0.9132, 0.9505]	[0.9392, 0.9630]	[0.9409, 0.9580]	[0.9342, 0.9651]	[0.8242, 0.8722]	[0.8747, 0.9329]	[0.7682, 0.9009]

Regarding computational efficiency of the 3 lightweight models, MambaOut-Femto exhibited the fastest inference time (7.85 ± 1.47 ms, 95% CI [6.02-9.68 ms]) with moderate GPU power consumption (43.43 ± 2.43 W, 95% CI [40.41-46.44 W]) and 6.15M parameters. MobileOne-S0, despite having the fewest parameters (4.27M) and lowest memory usage (59.6 ± 0.1 MB), required the longest inference time (29.03 ± 5.47 ms) but consumed the least GPU power (36.16 ± 1.06 W, 95% CI [34.84-37.48 W]). FastViT-S12 demonstrated balanced computational requirements with an inference time of 12.32 ± 1.84 ms and GPU power consumption of 41.03 ± 1.90 W, though it required more parameters (8.45M) and memory (188.2 ± 1.0 MB).

Additional lightweight model comparisons revealed MambaOut-Femto’s superiority. Compared to EfficientNet-B0, MambaOut-Femto achieved similar accuracy (91.06 ± 1.85% vs. 90.78 ± 2.41%) with faster inference (7.85 ± 1.47 vs. 10.19 ± 1.40 ms, 95% CI [6.02-9.68] vs. [8.46-11.93]) but higher GPU power (43.43 ± 2.43 vs. 35.88 ± 2.38 W, 95% CI [40.41-46.44] vs. [32.92-38.84]). Against ViT-Tiny, MambaOut-Femto demonstrated superior accuracy (91.06 ± 1.85% vs. 82.41 ± 4.06%, 95% CI [88.77-93.36] vs. [77.38-87.45]) and comparable GPU usage (43.43 ± 2.43 vs. 42.95 ± 3.49 W).

FLOPs analysis demonstrated exceptional efficiency among lightweight architectures. MambaOut-Femto, MobileOne-S0, EfficientNet-B0, and ViT-Tiny all required minimal computational resources at 0.010 GFLOPs, while FastViT-S12 needed 0.020 GFLOPs. Traditional architectures showed significantly higher computational demands, with ResNet50 requiring 0.050 GFLOPs and SwinV2-CR-Tiny demanding 0.060 GFLOPs, representing 5-6x higher computational overhead compared to lightweight models.

### 3.2 Model performance through stratified 5-fold cross validation

Performance evaluation was conducted using both single hyperparameter configuration and optimal hyperparameter tuning with 5-fold stratified cross-validation across two datasets. Under optimal configuration with stratified cross-validation, MambaOut-Femto consistently demonstrated superior performance across both datasets. On Dataset 1, MambaOut-Femto achieved the highest accuracy (89.62 ± 1.38%, 95% CI [87.90-91.33%]), precision (91.23 ± 1.12%, 95% CI [89.84-92.62%]), recall (91.21 ± 1.16%, 95% CI [89.77-92.65%]), F1-score (91.19 ± 1.17%, 95% CI [89.74-92.64%]), specificity (94.31 ± 0.76%, 95% CI [93.37-95.25%]), and AUC (97.20 ± 0.49%, 95% CI [96.59-97.82%]), while maintaining the lowest loss (0.2784 ± 0.0373, 95% CI [0.2321-0.3248]).

On Dataset 2, MambaOut-Femto maintained its superior performance with an accuracy of 91.57 ± 4.45% (95% CI [86.04-97.10%]), precision of 91.86 ± 5.07% (95% CI [85.56-98.16%]), and AUC of 96.80 ± 1.88% (95% CI [94.47-99.13%]). MobileOne-S0 showed consistent but slightly lower performance across both datasets, while FastViT-S12 demonstrated comparable results with slightly higher variability.

**Table 2** summarizes the mean, standard deviations, and 95% confidence intervals of five-fold cross-validation performance metrics for the final test set using the three models averaged across all folds for both datasets. MambaOut-Femto consistently outperformed the other models in terms of accuracy, precision, recall, F1-score, specificity, and AUC.

**Table 2:** Mean and standard deviations of five-fold cross-validation performance metrics for the final test set using the 3 models averaged across all folds for the two datasets.

Dataset	Metric	Mamba	MobileOne	FastViT
Dataset1	LOSS	0.2784 ± 0.0373 (95% CI [0.2321-0.3248])	0.3013 ± 0.0788 (95% CI [0.2034-0.3991])	0.3009 ± 0.0881 (95% CI [0.1915-0.410])
Dataset1	ACCURACY	0.8962 ± 0.0138 (95% CI [0.8790-0.9133])	0.8705 ± 0.0323 (95% CI [0.8304-0.9106])	0.8587 ± 0.0387 (95% CI [0.8106-0.9067])
Dataset1	PRECISION	0.9123 ± 0.0112 (95% CI [0.8984-0.9262])	0.8944 ± 0.0271 (95% CI [0.8607-0.9281])	0.8853 ± 0.0313 (95% CI [0.8465-0.9241])
Dataset1	RECALL	0.9121 ± 0.0116 (95% CI [0.8977-0.9265])	0.8908 ± 0.0272 (95% CI [0.8570-0.9246])	0.8814 ± 0.0326 (95% CI [0.8409-0.9218])
Dataset1	F1_SCORE	0.9119 ± 0.0117 (95% CI [0.8974-0.9264])	0.8897 ± 0.0274 (95% CI [0.8557-0.9238])	0.8796 ± 0.0331 (95% CI [0.8385-0.9208])
Dataset1	SPECIFICITY	0.9431 ± 0.0076 (95% CI [0.9337-0.9525])	0.9292 ± 0.0177 (95% CI [0.9072-0.9511])	0.9229 ± 0.0212 (95% CI [0.8967-0.9492])
Dataset1	AUC	0.9720 ± 0.0049 (95% CI [0.9659-0.9782])	0.9643 ± 0.0159 (95% CI [0.9446-0.9841])	0.9581 ± 0.0237 (95% CI [0.9287-0.9874])
Dataset2	Loss	0.3085 ± 0.1407 (95% CI: [0.1338, 0.4832])	0.3982 ± 0.0611 (95% CI: [0.3224, 0.4741])	0.3953 ± 0.2001 (95% CI: [0.1468, 0.6438])
Dataset2	Accuracy	0.9157 ± 0.0445 (95% CI: [0.8604, 0.9710])	0.8961 ± 0.0181 (95% CI: [0.8736, 0.9187])	0.8865 ± 0.0495 (95% CI: [0.8250, 0.9479])
Dataset2	Precision	0.9186 ± 0.0507 (95% CI: [0.8556, 0.9816])	0.9039 ± 0.0143 (95% CI: [0.8861, 0.9217])	0.9072 ± 0.0333 (95% CI: [0.8658, 0.9485])
Dataset2	Recall	0.9178 ± 0.0490 (95% CI: [0.8569, 0.9786])	0.9015 ± 0.0393 (95% CI: [0.8527, 0.9503])	0.9013 ± 0.0615 (95% CI: [0.8249, 0.9777])
Dataset2	F1 Score	0.9167 ± 0.0495 (95% CI: [0.8553, 0.9782])	0.8996 ± 0.0219 (95% CI: [0.8725, 0.9268])	0.9006 ± 0.0475 (95% CI: [0.8417, 0.9595])
Dataset2	Specificity	0.9499 ± 0.0254 (95% CI: [0.9184, 0.9813])	0.9359 ± 0.0170 (95% CI: [0.9147, 0.9570])	0.9288 ± 0.0366 (95% CI: [0.8834, 0.9742])
Dataset2	AUC	0.9680 ± 0.0188 (95% CI: [0.9447, 0.9913])	0.9573 ± 0.0161 (95% CI: [0.9373, 0.9774])	0.9565 ± 0.0317 (95% CI: [0.9172, 0.9958])

**Figure 2:**
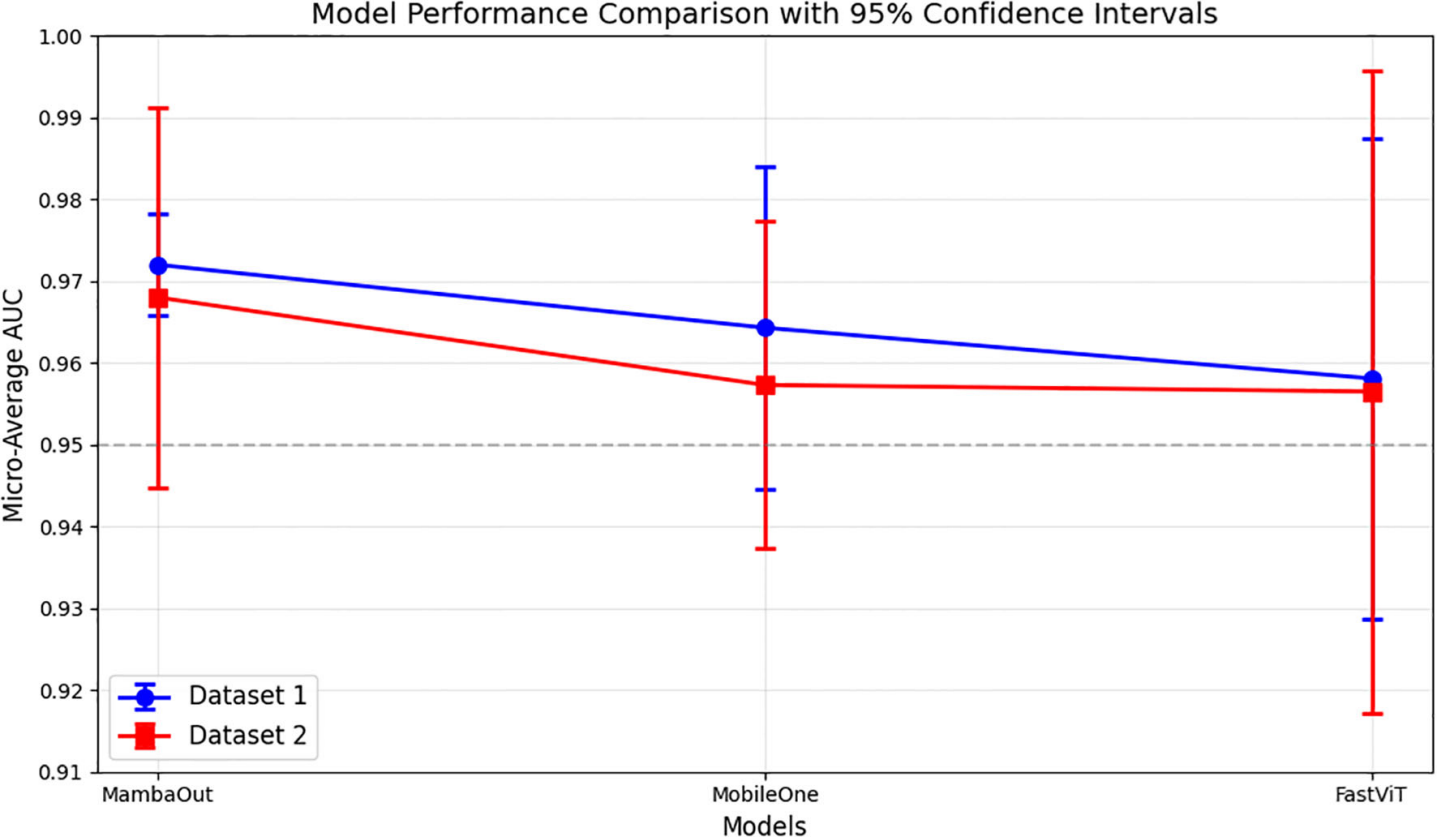
Micro-average AUC performance comparison of lightweight models across datasets. Results show mean AUC ± 95% confidence intervals from 5-fold cross-validation. Dataset 1, and Dataset 2.

The micro-average ROC curves from 5-fold cross-validation test sets for both datasets are shown in **Figure 2**. The results indicate that MambaOut-Femto achieved the highest AUC, followed by MobileOne-S0 and FastViT-S12.

**Figure 3:**
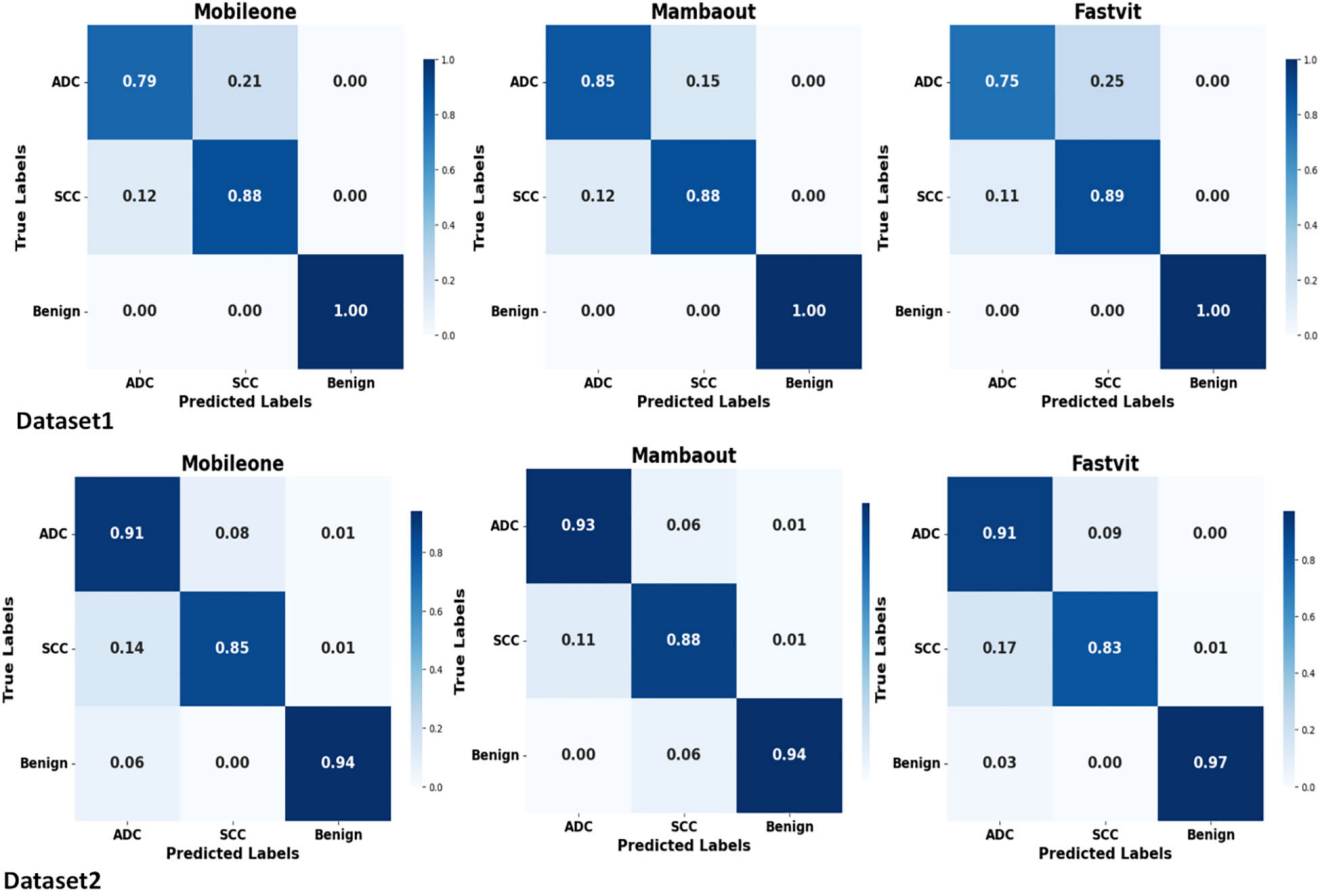
Average confusion matrices from 5-fold cross-validation test sets for MobileOne (I1), MambaOut, and FastViT.

**Figure 3** presents the average confusion matrices from 5-fold cross-validation test sets for MambaOut-Femto, MobileOne-S0, and FastViT-S12. The matrices highlight the models’ performance in classifying ADC, benign lesions, and SCC, with MambaOut-Femto showing the best overall classification performance.

The confusion matrices demonstrate balanced classification performance across all three classes (ADC, SCC, benign) with no evidence of majority class bias.

The results indicate that MambaOut-Femto is the most promising lightweight architecture for lung cancer CT scan image classification, offering a balance between performance and efficiency. MobileOne-S0 and FastViT-S12 also demonstrated strong performance, highlighting their potential for medical image classification tasks. MambaOut-Femto’s superior efficiency and performance metrics make it a suitable choice for real-world applications in medical imaging.

### 3.3 Best model visualization analysis

**Figure 4:**
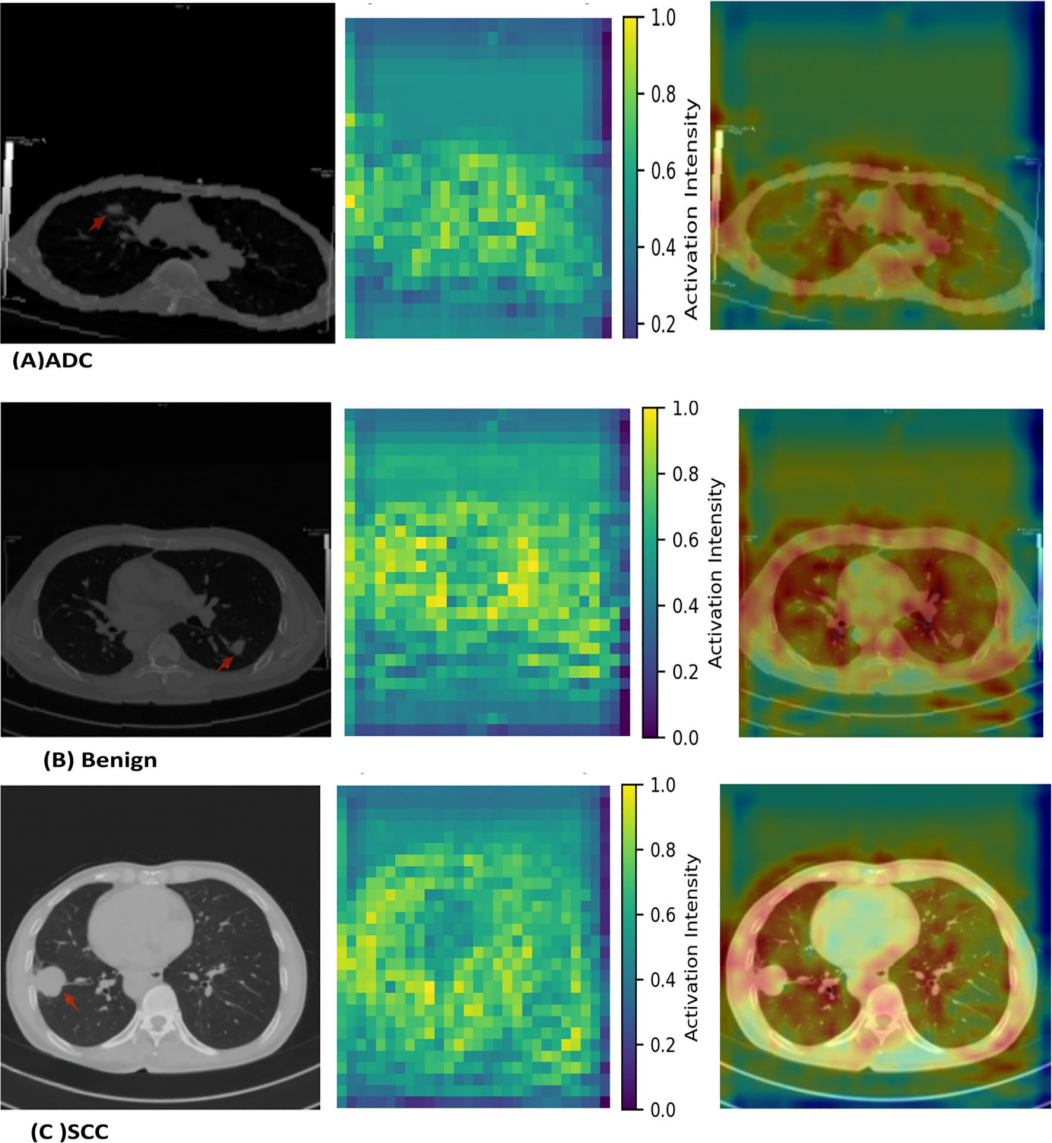
MambaOut-Femto activation patterns for lung cancer classification showing **(A)** adenocarcinoma, **(B)** squamous cell carcinoma, and **(C)** benign cases. Each panel displays the original CT scan, activation heatmap, and overlay visualization demonstrating class-specific attention patterns and correspondence between model focus and pathological features.

**Figure 4** demonstrates the interpretability of the best-performing MambaOut-Femto model through activation visualizations for each diagnostic class. The state-space model shows distinct activation patterns: malignant lesions (ADC and SCC) exhibit focused, high-intensity activation regions, while benign cases display distributed, lower-intensity patterns, confirming clinically relevant feature learning.

## 4 Discussion

Recent deep learning, especially using transformer-based models, has shown superior performance for the lung cancer classification task, as recent studies like Chen et al. (2024) (27) demonstrated significant progress using a volumetric SWIN Transformer, achieving 98.88% accuracy in distinguishing between benign nodules, adenocarcinoma, and squamous cell carcinoma. However, this approach requires substantial computational resources due to its 3D volumetric processing nature. When compared to recent transformer-based approaches like Huang et al.’s TBFE model (28) and Cao et al.’s multi-scale MobileViT (18), the proposed model achieves comparable or superior performance while maintaining a more lightweight architecture. This balance between performance and efficiency is particularly relevant for clinical integration, where computational resources may be limited.

The results of this study demonstrate the potential of lightweight pretrained models for lung cancer CT scan image classification. Among the evaluated models, MambaOut-Femto emerged as the most promising architecture, offering a balance between performance and efficiency. This section discusses the implications of these findings in the context of recent advancements and challenges in the field.

MobileOne-S0 and FastViT-S12 also demonstrated strong performance, highlighting their potential for medical image classification tasks. However, FastViT-S12 has the highest memory usage and a relatively higher inference time, which may limit its applicability in resource-constrained environments.

When compared to other studies in the field, the performance of these models is competitive. For instance, Attallah et al. (16) proposed a framework using ShuffleNet, MobileNet, and SqueezeNet models combined with feature reduction techniques, achieving a high accuracy of 99.6%. Similarly, Priya A & Shyamala Bharathi P (17). achieved 99.28% training accuracy and 98.03% testing accuracy using the EfficientNet model. The current study’s findings align with these results, indicating that lightweight models can indeed achieve high diagnostic accuracy.

The efficiency metrics of the models evaluated in this study are particularly noteworthy. MambaOut-Femto demonstrated the highest efficiency with a low inference time and a small number of parameters. This is advantageous for real-time applications and large datasets. In comparison, Klangbunrueang et al. (29) found that while VGG16 achieved a test accuracy of 98.18%, it required more computational resources than lighter models like MobileNetV2. The current study’s focus on efficiency highlights the potential for deploying these models in resource-constrained environments.

In our study, FastViT demonstrated superior performance in lung cancer CT scan image classification compared to SwinV2-tiny and ViT-tiny. This is consistent with the findings of Ko et al. (30), who evaluated FastViT alongside other vision transformer models for lung disease detection in chest X-ray images.

In the study by Ko et al. (30), FastViT achieved an accuracy of 97.63% with the NAdam optimizer on an imbalanced dataset. When compared directly to SwinV2-tiny and ViT-tiny in our study, FastViT showed higher test accuracy (0.9078 ± 0.0112) than SwinV2-tiny (0.7021 ± 0.0930) and ViT-tiny (0.8241 ± 0.0406). FastViT also demonstrated a better balance between performance and efficiency metrics, such as inference time and memory usage.

In the current study, MobileOne demonstrated impressive performance in lung cancer CT scan image classification, achieving a test accuracy of 0.8752 ± 0.0282 and AUC. While this is slightly lower than EfficientNet-B0’s 0.9078 ± 0.0241, MobileOne significantly outperformed traditional CNN models like ResNet-50, which achieved only 0.7234 ± 0.0365. What sets MobileOne apart is its ability to achieve this performance with a parameter count, and GPU power usage comparable to EfficientNet-B0 (4.27M vs. 4.01M), (36.16 ± 1.06, 35.88 ± 2.38 W) but with substantially lower memory usage (59.6 ± 0.1 vs. 215.8 ± 1.0 MB). This makes MobileOne a powerful choice when balancing performance and efficiency in CNN tasks, particularly in scenarios where memory resources are constrained. The model’s original design for mobile and edge devices translates well to medical imaging tasks, offering a viable alternative to traditional CNN architectures.

The Mamba model, introduced in late 2023 (15), has shown great potential in medical imaging for lung cancer detection. Since then, several variants have been developed:

-MedMamba (31) demonstrated high accuracy and low computational complexity for lung cancer detection in chest CT images, even without extensive pre-training.
- CT-Mamba (32) effectively reduced noise in low-dose CT images while enhancing detail preservation.
- Mamba network (33) improved pulmonary nodule detection by incorporating techniques such as deep separable convolution and spatial pyramid pooling.

The MambaOut model used in the current study offers a key advantage over these variants: it benefits from pretrained weights on the large ImageNet dataset. This allows it to be fine-tuned for specific medical tasks without training from scratch, saving computational resources and time. The pretrained weights enable MambaOut to generalize better and adapt more quickly to the features in lung cancer CT scans, making it highly suitable for clinical integration where efficiency and accuracy are crucial.

Recent lightweight architectures from computer vision have shown remarkable efficiency gains, yet their potential in medical imaging remains largely untapped. This study demonstrates that recently developed models offer distinct advantages for clinical deployment: MambaOut-Femto achieves superior accuracy with optimal inference speed, MobileOne-S0 provides the most parameter-efficient solution, while FastViT-S12 delivers balanced performance across metrics. By establishing these baseline comparisons, we provide healthcare decision-makers with empirical evidence for selecting appropriate models based on their specific computational constraints and performance requirements.

However, overlapping confidence intervals between configurations for several models highlight the importance of statistical validation before claiming clinical superiority. While improvements are consistent, they often fall within seed-induced variability ranges identified by Picard (2021) (34), emphasizing the need for rigorous statistical analysis in medical imaging applications where false conclusions carry high stakes.

This study has several limitations that warrant consideration. First, the generalizability of the models to other medical imaging datasets or different types of cancer is uncertain, as the study focused solely on lung cancer CT scans. Further validation on diverse medical imaging data is necessary to assess the models’ broader applicability. Second, while the models demonstrated strong performance metrics, their clinical applicability in real-world settings requires further investigation. Integrating these models into existing clinical workflows and evaluating their impact on diagnostic outcomes in practical scenarios is essential. Third, the interpretability analysis presented in this study is primarily qualitative, relying on visual inspection of activation patterns from randomly selected cases without quantitative validation metrics. While the Grad-CAM visualizations provide insights into model attention patterns, we did not perform quantitative localization assessments such as pointing-game scores or intersection-over-union calculations against expert annotations. Fourth, while radiologist annotations were available for the selected cases, quantitative correlation analysis between model attention patterns and expert annotations was not performed. Fifth, the relatively small sample sizes (n=95 for the public dataset, n=274 for the private dataset) may limit generalizability and pose a potential risk of overfitting despite cross-validation measures. Finally, the slice-level data partitioning may lead to optimistic performance estimates due to potential correlation between adjacent slices from the same patient, as slices from the same patient could appear in both training and test sets. While this approach follows established field practices and affects all compared models equally, it may limit the generalizability of absolute performance metrics to completely independent patient populations.

Future research should focus on enhancing the interpretability of these models through techniques such as saliency mapping or attention mechanisms, and quantitative correlation metrics between model attention and expert annotations with inter-rater agreement assessment. Implementation of quantitative localization metrics such as pointing-game scores, intersection-over-union calculations, and sensitivity/specificity measures for attention localization accuracy. This requires establishing ground truth through expert radiologist annotations with formal inter-rater agreement assessments using multiple readers and standardized annotation protocols.

Additionally, exploring the integration of these lightweight models with advanced technologies, such as federated learning or edge computing, could further enhance their performance and applicability in resource-constrained environments. Expanding the study to include multimodal imaging data and validating these findings on larger cohorts, such as the National Lung Screening Trial (NLST) and Lung Image Database Consortium (LIDC) datasets, to ensure robust generalization. Implementation of patient-level splitting when datasets provide patient-level annotations to ensure complete independence between training and test sets. Additionally, validation on larger, more diverse datasets with patient-level partitioning would provide more robust estimates of model performance in clinical settings.

Furthermore, assessing the models’ performance on different types of cancer and medical imaging modalities would broaden their potential impact in the medical field.

## 5 Conclusions

In conclusion, this work indicates the efficiency of lightweight pre-trained models for lung cancer CT scan image classification. MambaOut-Femto emerged as the most promising design, delivering a superior mix of performance and efficiency. This makes it a great alternative for real-world applications in medical imaging, particularly in circumstances with restricted processing resources. MobileOne-S0 and FastViT-S12 also showed strong performance, demonstrating their potential for medical image classification tasks. The results align with earlier research, demonstrating that lightweight models can achieve excellent diagnostic accuracy while minimizing computational complexity. Overall, these findings imply that lightweight models have the potential to greatly impact medical imaging, delivering efficient and accurate solutions for lung cancer diagnosis. Further study and clinical validation are needed to fully understand their benefits in practical applications.

## Data Availability

Patient CT data from Institution 1 cannot be shared publicly; access requires institutional approval. Dataset 2 (Zenodo) is public. Code is available on request.
