# Correction: Evaluation of recent lightweight deep learning architectures for lung cancer CT classification

**DOI:** 10.3389/fonc.2025.1715188

**Published:** 2025-10-21

**Authors:** MennaAllah Mahmoud, Yanhua Wen, Xiaohuan Pan, Yuling Liufu, Yubao Guan

**Affiliations:** ^1^ Radiology Department, The Fifth Affiliated Hospital of Guangzhou Medical University, Guangzhou, China; ^2^ Radiology Department, First Affiliated Hospital of Guangzhou Medical University, Guangzhou, China

**Keywords:** deep learning, pre-trained models, lightweight models, lung cancers, computed tomography

Author “Yanhua Yen” was erroneously omitted as equal contributing author. The original version of this article has been updated.

The correct author list reads:

“MennaAllah Mahmoud^1*†^, Yanhua Wen^1†^, Xiaohuan Pan^2^, Yuling Liufu^1^ and Yubao Guan^1*^”

The original version of this article has been updated.

